# Platelet-rich plasma improves therapeutic effects of menstrual blood-derived stromal cells in rat model of intrauterine adhesion

**DOI:** 10.1186/s13287-019-1155-7

**Published:** 2019-02-15

**Authors:** Siwen Zhang, Pingping Li, Zhengwei Yuan, Jichun Tan

**Affiliations:** 1 Key Laboratory of Reproductive Dysfunction Diseases and Fertility Remodeling of Liaoning Province, Reproductive Medicine Center, Obstetrics and Gynecology Department, Shengjing Hospital affiliated to China Medical University, No. 39 Huaxiang Road, Tiexi District, Shenyang, Liaoning China; 2 Key Laboratory of Health Ministry for Congenital Malformation, Shengjing Hospital affiliated to China Medical University, No. 7, Economic Development Zone, Benxi, Liaoning China

**Keywords:** Intrauterine adhesion, Rat model, MenSCs, PRP

## Abstract

**Background:**

Intrauterine adhesion (IUA) is a major cause of female secondary infertility. We previously demonstrated that menstrual blood-derived stromal cell (MenSC) transplantation helped severe IUA patients have pregnancy and endometrium regeneration. We also initiated platelet-rich plasma (PRP) acted as a beneficial supplement in MenSC culturing and a potential endometrial receptivity regulator. Here, we investigated the therapeutic effect of combined transplantation of MenSCs with PRP in rat IUA models and the mechanisms of MenSCs in endometrium regeneration.

**Methods:**

Rat IUA models were established by intrauterine mechanical injured. Nine days later, all rats were randomly assigned to four groups received different treatment: placebo, MenSC transplantation, PRP transplantation, and MenSCs + PRP transplantation. The traces of MenSCs were tracked with GFP label. Endometrial morphology and pathology, tissue proliferation, inflammation, pregnancy outcomes, and mechanism of MenSCs in the regeneration of endometrium were investigated.

**Results:**

Notably, at days 9 and 18 post-treatment, MenSC transplantation significantly improved endometrial proliferation, angiogenesis, and morphology recovery and decreased collagen fibrosis and inflammation in the uterus. MenSCs had lesion chemotaxis, colonized around the endometrial glands. Gene expression of human-derived secretory protein *IGF-1*, *SDF-1*, and *TSP-1* was detected in the uterus received MenSCs at day 18. The three treatments can all improve fertility in IUA rats. Moreover, gene expressions of cell proliferation, developmental processes, and other biological processes were induced in MenSC transplantation group. Hippo signaling pathway was the most significantly changed pathway, and the downstream factors CTGF, Wnt5a, and Gdf5 were significantly regulated in treatment groups. PRP enhanced these parameters through a synergistic effect.

**Conclusions:**

In summary, MenSCs could effectively improve uterine proliferation, markedly accelerate endometrial damage repairment and promote fertility restoration in IUA rats, suggesting a paracrine restorative effect and Hippo signaling pathway stimulation. Our results indicate MenSCs, a valuable source of cells for transplantation in the treatment intrauterine adhesion. Combined with PRP, this cell therapy was more effective.

**Electronic supplementary material:**

The online version of this article (10.1186/s13287-019-1155-7) contains supplementary material, which is available to authorized users.

## Background

Intrauterine adhesion (IUA) is a major cause of female secondary infertility [1]. The classical therapeutic approaches for infertile patients with IUA are hysteroscopic adhesiolysis, removing visible intrauterine adhesions. Postoperative placement of intrauterine devices (IUD), gel intrauterine injection, and estrogen administration are advised to prevent adhesion recurrence and to enhance endometrial proliferation [2, 3]. Nevertheless, in some cases with severe endometrial injuries, it is hard to get repaired, even using surgical operation combined with hormone treatment, resulting in poor prognosis [4].

Damage to the basal layer of endometrium is the primary cause of IUA. Since the functional layer undergoes degeneration, growth, and reorganization throughout the menstrual cycle, in which the process depends on the intact basal layer, the existence of endometrial stem cells in the basalis was postulated. Several studies have demonstrated the existence of an adult stem cell population capable of regenerating endometrial tissue, although its circulatory origin cannot be ruled out [5, 6]. Thus, supplementing stem cells from the uterus or other tissues is one of the research hotspots for endometrium regeneration. With their capability to integrate into the injured tissues, bone marrow-derived stem cells (BMSCs) are commonly used for cell therapy [7, 8]. BMSCs could increase the endometrial thickness and restore tissue morphology, thus improving uterine receptivity in some subjects [8]. Nevertheless, the main restriction for the application of BMSCs is its invasiveness of collection. Thus, a less invasive source of stem cell needs to be investigated.

Several studies have suggested that menstrual blood-derived stromal cells (MenSCs) are comparable to BMSCs in treating some diseases [9]. MenSCs have high proliferative and self-renewal capacities and can be easily obtained through a painless procedure without ethical concerns [10]. Therefore, in our previous clinical study, we have explored the therapeutic efficacy of MenSCs in patients with severe IUA [11]. A significant increase of endometrial thickness was found in all subjects, and some women successfully became pregnant after frozen embryos transfer. However, the long recovery time and ongoing pregnancy rates were still not optimal.

Platelet-rich plasma (PRP) is an autologous plasma product with platelet concentrations above baseline values, and PRP releases several growth factors including vascular endothelial growth factor (VEGF), platelet-derived growth factor (PDGF), insulin-like growth factor (IGF), basic fibroblast growth factor (b-FGF), and transforming growth factor-β1 (TGF-β1) that are all beneficial for wound healing. Multiple trials have explored the application of PRP in acute and chronic injuries [12, 13]. The most common use of PRP is for skeletal damages, whereas sporadic studies have shown its effectiveness in treating mucous membrane and skin defects [14, 15]. A co-culture study suggested that PRP significantly improved stem cell viability and could be used as an autologous cell delivery system in repairing tissue damage [16]. Our recent work showed that PRP could promote the proliferation of MenSCs. Remarkably, it improves cell stemness, angiogenesis, and expression of receptivity markers in vitro [17].

Thus, in this study, we aimed to evaluate whether combined transplantation of MenSCs with PRP is more effective in endometrial regeneration in vivo. Furthermore, we investigate the underlying mechanism of MenSCs in the restoration of endometrium function.

## Methods

### Ethical approval

This study was approved by the Ethics Committee of the Shengjing Hospital affiliated to China Medical University (2017PS330K) and has been performed in accordance with the principles of the Declaration of Helsinki.

### MenSC culture

Three menstrual blood donors were diagnosed without reproductive system diseases, aged 25, 25, and 27 years old. All donors gave consent, and all procedures were approved by the Ethics Committee of Shengjing Hospital affiliated to China Medical University (2017PS330K).

The culture and identification of MenSCs were performed in the same way as our previous article [17]. Briefly, menstrual blood was sterilely collected and isolated. The cells were regularly cultured in DMEM/F12 medium (1:1, HyClone, Logan, UT, USA) with 10% fetal bovine serum (Gibco, Waltham, MA, USA ) under 37 °C in an atmosphere containing 5% CO_2_. The culture medium was changed every 3 days until adherent cells reach 80–90% confluence, then the cells were passaged by 0.25% trypsin (Sigma-Aldrich). P3 MenSCs were collected and measured surface markers of mesenchymal stem cell. MenSCs were transplanted before the sixth passage.

### PRP preparation

PRP was extracted from apheresis platelets (with a concentration of 12.5 × 10^11^/L, acquired from the blood transfusion department of Shengjing Hospital) according to our previous article [17]. For activation, 20% CaCl_2_ (Sigma-Aldrich) plus 1000 U/ml thrombin (T8020, Solarbio, Beijing, China) was added at a volume ratio of 1:20. The mixture was incubated at 37 °C for 1 h and at 4 °C for 12 h. After activation, the gel-like mixture was centrifuged, and the supernatant was aspirated and filtered through 0.22-μm filters stored at − 80 °C.

### Rat IUA model

Female Sprague-Dawley rats, at 10 weeks of age, were purchased from HFK Bioscience Co. (Beijing, China). The rats were housed in SPF (specific pathogen-free) lab (SYXK 2017-0004, China) in an environment with a temperature of 22 ± 1 °C, relative humidity of 50 ± 1%, and a light/dark cycle of 12/12 h. Sterilized food and water were available ad libitum. All animal studies (including the euthanasia procedure) were done in compliance with the regulations and guidelines of China Medical University institutional animal care and conducted according to the AAALAC and the IACUC guidelines.

After a week to achieve regular estrous cyclicity determined by vaginal smearing, the rats were anesthetized by 1 ml/kg 3% sodium pentobarbital i.p. on diestrus. Then, a vertical incision (~ 20 mm) was preformed longitudinally, followed by the insertion and rotation of a 16-gauge needle to scratch the entire inner endometrial surface of both laterals, until the uterine walls became rough and pale, leaving the uterine serosa intact. The uterus was subsequently washed with sterile normal saline. Finally, the uterine and abdominal wounds were closed.

### GFP labeling of MenSCs

Stocks of replication-defective adenoviral (Genechem, China) vectors expressing the green fluorescent protein (GFP) were stored at − 80 °C. P2-P3 MenSCs were seeded in a 60-mm culture dish to ~ 30% confluence. After adherence for 6 h, cells were infected (MOI = 20) with adenovirus-GFP in 10 μg/ml polybrene and incubated at 37 °C for 12 h, followed by a change with fresh medium. Four days later, GFP expression was analyzed under a fluorescence microscope.

### Treatment of rat IUA model

After two estrous cycle (~ 9 days), the established IUA model rats were randomly divided into four groups: placebo PBS injection group (N group), 5 × 10^5^ MenSCs per uterus suspended in PBS injection group (M group), PRP injection group (P group), and 5 × 10^5^ MenSCs per uterus suspended in PRP injection group (MP group). All animals were anesthetized and underwent laparotomy to expose the uterus. The total amount of fluid received was 50 μL per uterus, injected into the uterine subserosin with 32 G syringe. The rats were sacrificed 9 or 18 days after treatment.

### Bioluminescence imaging (BLI)

To locate cell homing visually, GFP-MenSCs were injected into the subserosa of the right lateral uterus of model rats. Eighteen days after injection, six animals were sacrificed. The uteri were removed together with the ovaries, liver, and brain. Subsequently, each tissue was placed into a Petri dish and transferred onto the scanning stage of the in vivo MS FX Pro system (Carestream, USA) for image acquisition. BLI was carried to identify the location of GFP-MenSCs in the tissues. Images were acquired and analyzed by Carestream MI SE software.

### Rats’ mating

Since 18th day of treatment, the female rats were housed together with healthy fertile male rats at a ratio of 2:1. The mating attempt was repeated every day at 9 pm until the sperm was found in a vaginal smear on the next morning, which was recorded as E0.5. The pregnant female rats were sacrificed on E13.5, the gestational sacs were counted, and the embryo development was photographed.

### Histological analysis

Specimens of the uteri were fixed in 4% neutral-buffered formalin, dehydrated, and embedded in paraffin. Serial sections of 5 μm were prepared and stained with hematoxylin and eosin (H&E), according to Ourchem (China, Cat#71020784) and SCR (China, Cat#71014544) application procedures. Masson trichrome (Solarbio, China, Cat#G1340) staining was applied to detect fibrosis according to manufacturer’s protocol. Undamaged uteri were used as negative control. No less than six uterine cross sections were evaluated for each group, and each section was randomly selected from three locations to measure or score. The endometrial thickness was measured using ImageJ software. The amount of fibrosis (blue) was scored as follows: 0—no blue, 1—minimal blue, 2—moderate blue, and 3—dense blue. The number of the vessels was evaluated as the number of the capillary vessels counted under a magnification of × 100.

### Immunohistochemistry

To identify scar formation or key protein expression by immunohistochemistry, uteri sections were transferred into adhesive slides and dried at 60 °C for 2 h. All slides were de-paraffinized using xylene and then rehydrated in decreasing concentrations of ethanol. Antigen retrieval was performed using microwave heating (two times for 5 and 8 min; 10-mM citrate buffer, pH 6.0). After inhibition of endogenous peroxidase for 15 min and protein block for 30 min at 37 °C (ZSGB-BIO, Cat#SP-9001), the slides were incubated with rabbit polyclonal antibodies to collagen I (1:200; Abcam, UK, Cat#ab34710), anti-vimentin (1:100; Wanleibio, China, Cat#WL01960), or anti-Cytokeratin 18 (1:200; Abcam, UK, Cat#ab181597) overnight at 4 °C, then washed using PBS and incubated with secondary antibody for 2 h at room temperature followed by PBS wash. The primary antibody was replaced by PBS as negative controls. Finally, the detection of bound antibody was accomplished using the avidin-biotin complex reagent for 20 min followed by PBS wash. A 0.1% solution of diaminobenzidine was used for 2 min as a chromogen. Slides were counterstained with Mayer’s hematoxylin for 2 min. Staining intensity was scored as 0 (negative), 1 (weak), 2 (medium), or 3 (strong).

### Immunofluorescence

To detect cell proliferation in the endometrium, 48 h before rats were sacrificed, 5-μL EdU (5-ethynyl-2′-deoxyuridine, a thymidine analog) solution dissolved in 1-ml normal saline was injected (i.p.). Then, EdU staining of uterine slides was performed according to manufacturer’s protocol (Ribobio, China, Cat#C10310). The slides were then incubated with DAPI solution (KeyGEN Biotech, China, Cat#KGA215) for nuclear staining. The slide from normal mice without EdU injection was stained as a negative control. The immunofluorescence was captured by the Nikon Eclipse Ni (Nikon, Tokyo, Japan), the number of red- and blue-staining cells was counted and averaged from at least 3 randomly selected fields under a magnification of × 400. The positive rate (PR) was analyzed by Image-Pro^®^ Plus 6 (Media Cybernetics Rockville, MD, USA), calculated according to the following formula: PR = EdU^+^/DAPI^+^*100%.

To locate the transplanted MenSCs, sections from uteri were blocked with PBS (pH 7.4) and 3% dehydroxide solution for 5 min and then with blocking solution (Invitrogen, US) for 30 min before being incubated with the primary antibodies including anti-GFP (1:2000; Abcam, UK, Cat#ab13970) and anti-PDGFRβ (1:100; Abcam, UK, Cat#ab32570). After incubation for 90 min with the primary antibodies at 4 °C, the sections were washed with PBS again before being incubated with secondary antibody (Cy3-labeled goat anti-mouse IgG, 1:300, Beyotime, China, Cat#A0521, or Cy3-labeled goat anti-rabbit IgG, 1:300, Beyotime, China, Cat#A0516) for 90 min at room temperature. Subsequently, nuclear staining was conducted with DAPI. Tissues were evaluated with confocal laser scanning microscopy (Nikon ECLIPSE N80-i). Image analysis was done using Image-Pro^®^ Plus 6 software.

### Real-time PCR

Total RNA were extracted from excised uterine tissues by using the RNAiso Plus kit (Takara, China, Cat#9108). The purity and concentration of RNA were verified by NanoDrop 2000 spectrophotometery (NanoDrop Technologies, Thermo Scientific). Two micrograms of total RNA was reverse transcribed to generate cDNA (Takara, China, Cat#RR047A). Quantitative real-time PCR using 96-well optical plates was performed in a SYBR Green (Takara, China, Cat#RR820A) format with 20 μL template and analyzed with 7500 Fast Real-Time PCR Detection Systems (Life Technology), each sample was done in triplicate. Primer sequences used for each target gene are summarized in Additional file 1: Table S1. The cycling parameters for the PCR were as follows: initial denaturation at 95 °C for 30 s followed by 40 cycles of 5 s at 95 °C and 34 s at 60 °C. Analyses of relative gene expressions were performed using 2^−ΔΔCT^ methods. Ratios of mRNA expressions were given as fold-changes relative to untreated controls after normalizing to allogeneic *Gapdh* housekeeping gene.

### LUMINEX assay

The supernatant of uterine homogenate was collected and evaluated in the Luminex immunobead platform using kits according to manufacturer’s recommended protocols (Thermo Fisher Scientific, Waltham, USA). Inflammatory cytokine panels of IL-1β, IL-4, IL-6, and IL-10 were analyzed.

### RNA sequencing

After integrity and concentration testing for total RNA of the uterus from N group, M group, and MP group, the mRNAs were enriched with each sample using Oligo(dT)-magnetic beads. Fragmentation buffer was added to mRNA samples to make RNAs into short fragments, and then, the amplified mRNA was used as a template to synthesize first-strand cDNAs with a six-stranded random primer. Second-strand cDNAs were synthesized by adding the buffer, dNTPs, RNase, and DNA polymerase I. The double-stranded cDNAs, purified by QIAquick PCR kit and eluted with Buffer EB, were subjected to terminal repair and addition of sequencing adapters, followed by agarose gel electrophoresis to recover the target fragment for PCR amplification. The complete library was sequenced with Illumina HiSeq X Ten PE150 sequencing strategy.

The sequence reads from transcriptome sequencing were aligned to the Rattus norvegicus genome by HISAT2 with default parameters. In RNA-Seq, the relative expression of a transcript is proportional to the number of cDNA fragments that originate from it. The formula of FPKM (fragments per kilobase million) is FPKM = 10^9^ × *F*/*NL*. If FPKM (A) is the expression level of gene A, then *F* is the fragment number with a unique alignment to the gene A, *N* is the total fragment number with a unique alignment to the reference gene, and *L* is the length of the exon of the gene A. The calculated gene expression value was directly used for comparing the gene expression differences in different samples. To further understand the biological functions influenced by different experimental conditions, differential expression analysis was performed in the control group and the treatment group using R package DESeq2 with the criterion of |log_2_foldchange| ≥ 1 and *q* < 0.05. To better understand the commonalities of the differentially expressed genes in each comparison group, a Venn diagram was made. In order to understand the biological functions affected, differentially expressed genes in each comparison group were enriched to identify statistically overrepresented GO terms and biological pathways. KOBAS was applied to identify significantly enriched pathways using hypergeometric test and Benjamini-Hochberg FDR correction method with corrected *P* < 0.05.

### Western blotting

The uterine homogenate was prepared using RIPA (Beyotime, China, Cat#P0013B) with PMSF (Beyotime, China, Cat#ST506). Protein concentration was quantified using BCA kit (Beyotime, China, Cat#P0010S). Aliquots of protein samples (10 μg) were separated by SDS-PAGE (Beyotime, China, Cat#P0012A). The separated proteins were subsequently blotted on to PVDF membrane (Millipore, Billerica, MA, USA) and incubated with primary antibodies against Wnt5a (1:500, Wanleibio, China, Cat#WL02186), Ctgf (1:500, Wanleibio, China, Cat#WL02602), Gdf-5 (1:500, Absin, China, Cat#abs126292a), and Gapdh (1:1000, GOODHERE, China, Cat#AB-P-R-00). The membrane was then washed and treated with peroxidase-conjugated secondary AffiniPure goat anti-rabbit antibody (1:5000, Zsgb-Bio, China, Cat#ZB2301), processed using enhanced chemiluminescence reagents (Beyotime, China, Cat#P0018), and scanned with a Darkroom Eliminator (Azure Biosystems, USA, Cat#C300).

### Statistical analysis

One-way analysis of variance (ANOVA) was used to analyze study variables among rat groups, and Bonferroni post hoc tests were performed for multiple comparisons when statistical significance was recognized. All numerical values are presented as the mean ± standard deviation (SD), and *P < 0.05, **P < 0.01, and ***P < 0.001 were considered to indicate a statistically significant difference. All statistical analyses were performed using GraphPad Prism 5 software (San Diego, CA, USA).

## Results

### Location of MenSCs in the endometrium in rat IUA model

**Fig. 1:**
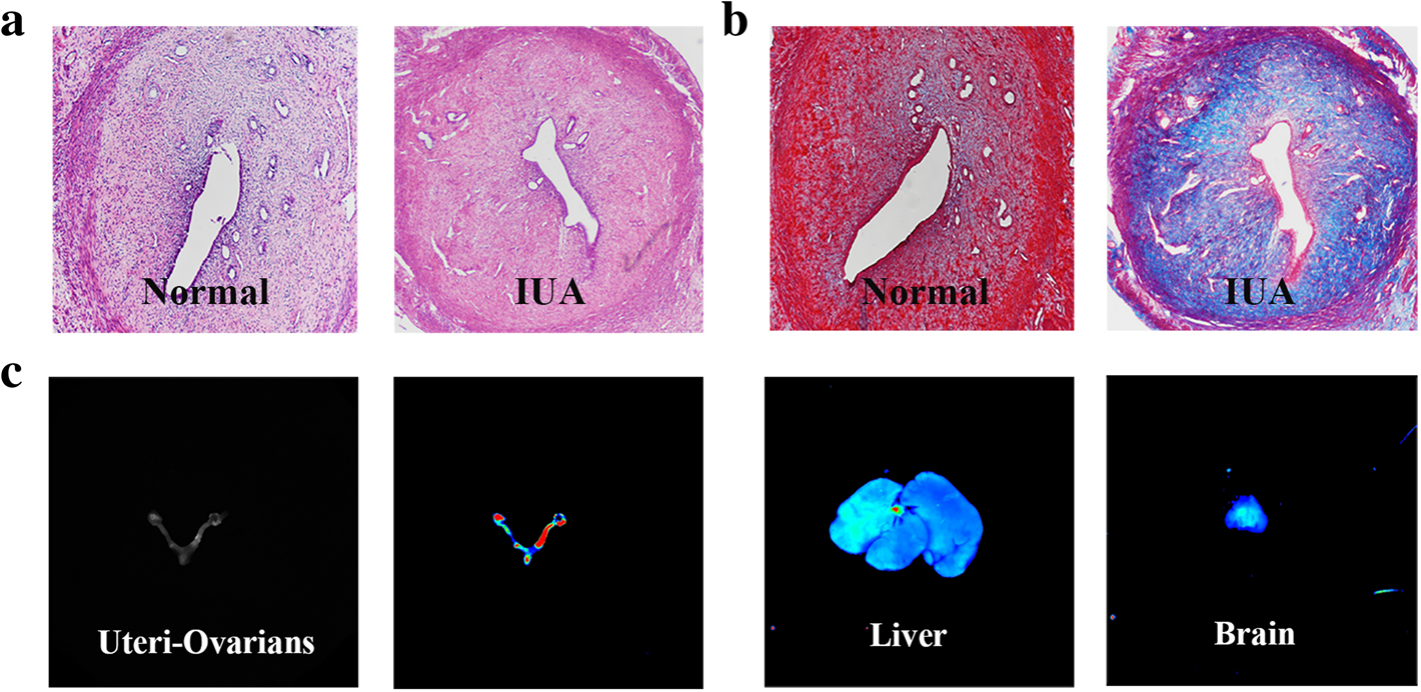
Establishment of rat IUA model and the location of GFP-MenSCs after transplantation. **a** H&E staining of the uterus from a normal rat (left) and model rat (right) at day 9 after damage. **b** Masson staining of the uterus from a normal rat (left) and model rat (right) at day 9 after damage (× 100). **c** The location of GFP-MenSCs in the uteri, ovary, liver, and brain after injected into the uterus at day 18. Blue, low fluorescence intensity; red, high fluorescence intensity

Due to the ethical restriction related to the human specimen, rat IUA model was established according to previous reports to clarify the mechanism underlying MenSCs and PRP transplantation in endometrium restoration [18, 19]. After uterine scratching, H&E staining (*n* = 4) showed discontinuous luminal surface, reduced glands in the basalis of damaged uteri, and loss of luminal cavity (Fig. 1a). Masson staining (*n* = 4) displayed significantly increased fibrosis in the uteri (Fig. 1b). Consequently, modeled rats were randomly divided into four groups: N group, M group, P group, and MP group. To trace the location of injected cells, MenSCs was transfected with GFP. Positive-labeled MenSCs were verified by using a fluorescence microscope (Additional file 2: Figure S1). Eighteen days after injection of MenSCs (*n* = 6) into the right lateral uterus, a clear fluorescent signal was observed in bilateral uteri and ovaries, while the signal was rarely seen in other major organs including the brain, heart, and liver. The transferred MenSCs were integrated to the entire genital tract, indicating its potential to restore female fertility. Local cluster in the major vessel of hepatic hila also demonstrated the ability for long-distance migration of MenSCs in general circulation (Fig. 1c). Furthermore, at day 9 of injection, DiI-labeled MenSCs migrated into different layers of the endometrium (Additional file 3: Figure S2).

### Pregnancy outcomes of model rats after treatment

**Fig. 2:**
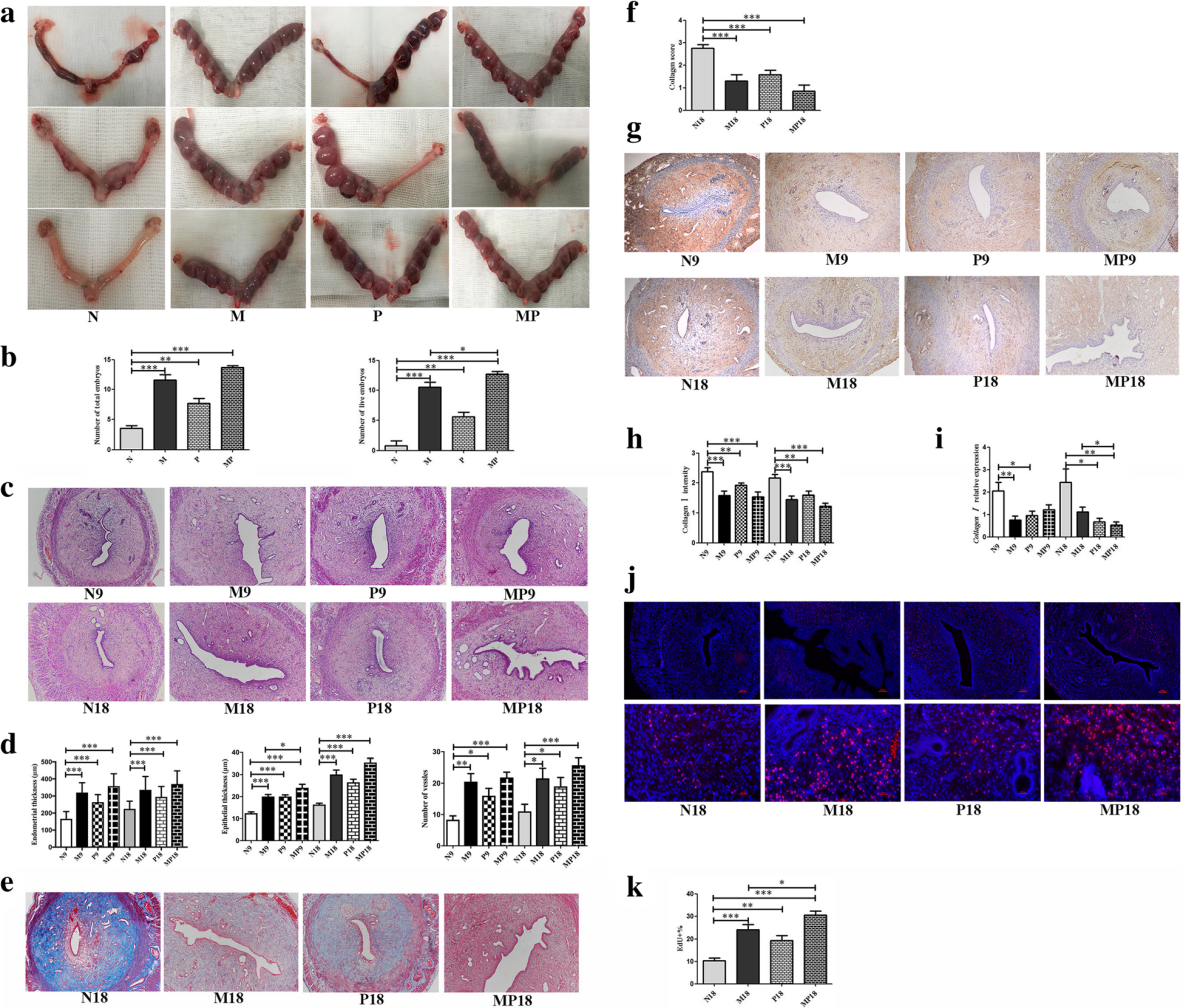
Pregnancy outcome, histological analysis, collagen deposition and cell proliferation in four treatment groups. **a** Rat uterus after 13.5 days of embryo implantation (E13.5). **b** Statistical analysis of total embryos and live embryos (N≥5). **c** Representative H&E staining of uterus at day 9 and day 18 (9- treatment for 9 days,18- treatment for 18 days, 100×). **d** Comparison of endometrial thickness, epithelial thickness and total vessel numbers at day 9 and day 18 (N≥6).**e** Representative Masson staining of uterus at day 18 (100×). **f** Comparison of collagen score at day 18 (N≥6). **g** Representative immunohistochemical staining of Collagen I at day 9 and day 18 (100×). **h** Comparison of Collagen Iintensity (N≥6). **i** Comparison of Collagen ImRNA expression at day 9 and 18 (N≥6). **j** EdU staining (red) of uterus at day 18, the nuclear was stained by DAPI (blue), upper panels (100×), lower panels (400×). **k** Comparison of EdU+ cell rate at day 18 (N≥6)

To check the functional improvement of the damaged uteri after MenSCs and/or PRP transplantation, female rats were bred starting on the 18th day after transplantation. Fetuses within the whole uteri were harvested on E13.5. As shown in Fig. 2a, the transplantation of MenSCs resulted in average 11.5 implantation sites, compared to only 3.6 gestational sacs in the damaged N group. Further analysis of viable fetuses with heart beats showed that almost none viable embryos could be found in uteri of N group. In contrast, MenSCs or PRP transplantation significantly promoted embryo implantation and subsequent development. MenSCs had a significantly stronger effect on improvement of fertility than PRP (*P* = 0.0006). Furthermore, the combination of MenSCs and PRP transplantation demonstrated the best outcomes (MP vs M group, *P* = 0.0372) (Fig. 2b).

### Effects of MenSCs and PRP in the restoration of endometrium damage

To identify the function of MenSCs and PRP in damage recovery of the endometrium, H&E staining was used to analyze endometrial thickness and vascularization. Significantly increased endometrial thickness was found after MenSCs or PRP or combination transplantation. Moreover, the epithelial thickness in MP group was significantly thicker than in M group at day 9. Compared to control N group, significantly increased vessel number was seen in M, P, and MP group at day 9 and day 18 after treatment (Fig. 2c, d). Similar effects between M and MP group implied that MenSCs had an important role in angiogenesis while PRP had a weaker effect. As collagen-producing fibroblast was recognized as an essential step for progressive fibrosis, Masson staining was performed in each group. Significant reduction of collagen deposition was found in M group, P group, and MP group at day 18 (Fig. 2e). Compared with M group, less fibrosis was observed in the MP group which suggested a trend toward significance (*P =* 0.0401) (Fig. 2f). Accordingly, the expression of collagen I was examined using real-time PCR and immunohistochemistry. As expected, MenSC and PRP treatment significantly decreased mRNA expression and deposition of collagen I at day 9 and day 18 (Fig. 2g–i). There was no difference between M group and MP group while significant lower protein intensity was found in MP group compared to P group at days 9 and 18 (*P =* 0.0196 and *P =* 0.0269), demonstrating that combination with MenSCs had a significantly stronger effect on reducing adhesions formation compared to PRP.

**Fig. 3:**
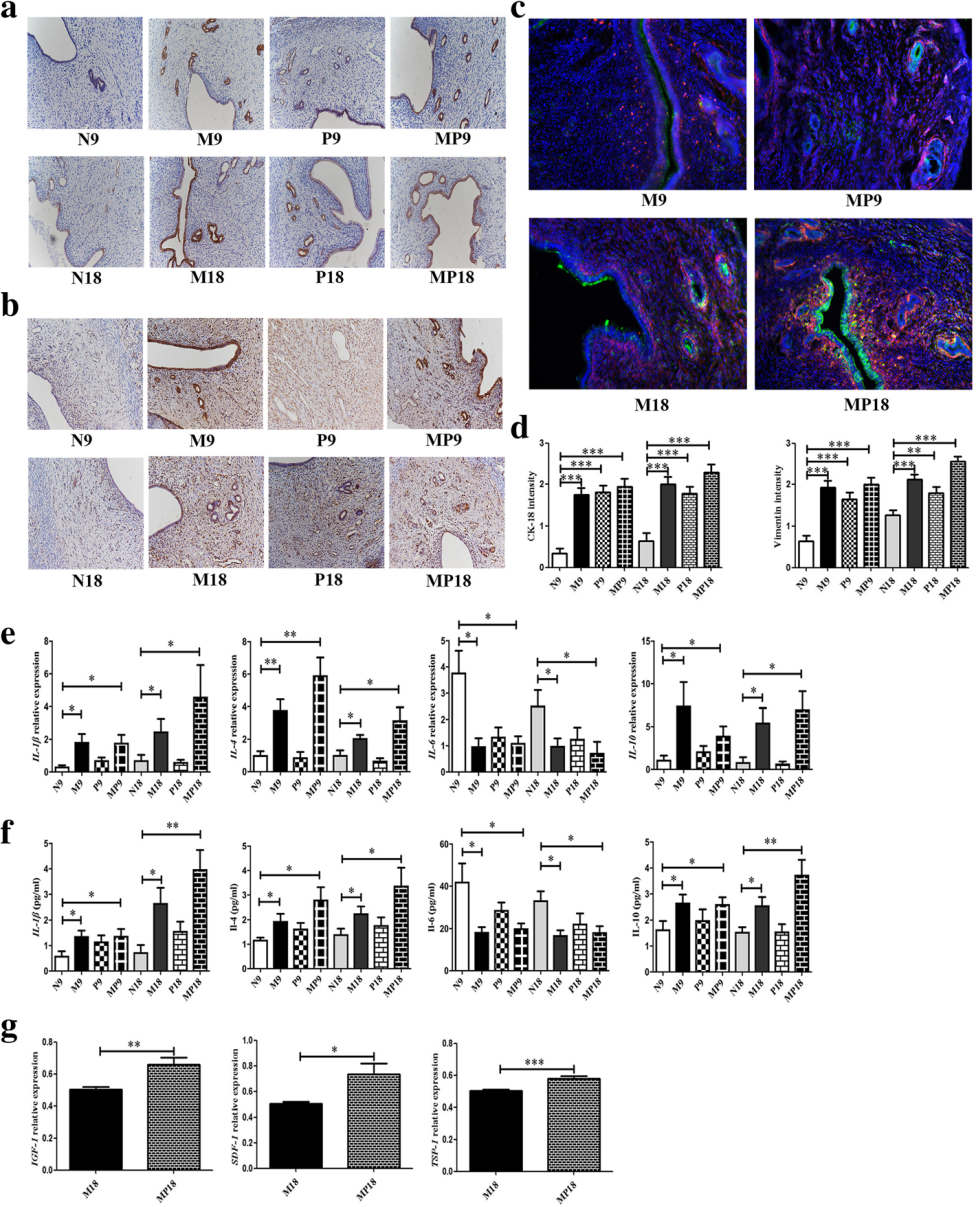
Analysis of markers of endometrium, inflammation and MenSCs secretion in uterus of four treatment groups. **a** Representative immunohistochemical staining of CK-18 in uterus at day 9 and day 18 (200×). **b** Representative immunohistochemical staining of Vimentin in uterus at day 9 and 18 (200×). **c** Immunofluorescent staining of GFP (green) and PDGFRβ (red) in uterus of M group and MP group at day 9 and day18. Nucleus was stained with DAPI (blue), while yellow staining indicated the co-expression of GFP and PDGFRβ (200×). **d** Comparison of CK-18 and Vimentin intensity at day 9 and day 18 (N≥6). **e** Real-time PCR analysis of rat genes including IL-1β, IL-4, IL-6and IL-10in uterus (N≥6). **f** LUMINEX analysis of rat IL-1β, IL-4, IL-6 and IL-10 in uterus(N≥6). **g** Real-time PCR analysis of human genes includingIGF-1,SDF-1and TSP-1between M group and MP group at day 18, data was normalized to human GAPDH (N=8)

Next, cell proliferation was evaluated using EdU staining. At day 18 post-treatment, a statistically significant increase of EdU+ cells was found in the epithelium and stroma of the traumatized uteri in M group, P group, and MP group compared to control N group. In comparison with M group, MP group showed significantly more proliferation which revealed a synergistic effect of PRP and MenSC transplantation (Fig. 2j, k). Furthermore, the regeneration of endometrium epithelial and stromal cells was evaluated by examining the expression of CK-18 (cytokeratin-18) and Vimentin, respectively, with immunohistochemistry. As shown in Fig. 3a, b, and d, at day 9 and day 18 post-translation, significantly more CK-18+ cells were observed in M group, P group, and MP group compared to control N group. Similarly, the expression of vimentin was also increased after treatment with MenSCs or PRP at days 9 and 18, while MenSCs demonstrated stronger function compared to PRP, and a synergistic effect was found in MP group. As the surface marker of endometrial perivascular stromal cells and previously shown to be a source of human endometrial mesenchymal stem cells, the expression of PDGFRβ in the uteri after transfer of GFP-MenSCs was detected in M group and MP group. GFP was found to be expressed in luminal epithelium, glandular epithelium, and basalis. PDGFRβ was mainly localized in the basalis. The strong yellow staining in the uterus of MP group at day 18 demonstrated that PRP significantly promoted human MenSC (GFP+PDGFRβ+) proliferation or survival after transplantation (Fig. 3c).

Inflammation of IUA was assessed in the uterus tissue using real-time PCR and LUMINEX. As shown in Fig. 3e, gene expressions of *IL-1β*, *IL-4*, and *IL-10* were increased, and *IL-6* was decreased in all treatment groups. As shown in Fig. 3f, LUMINEX for IL-1β, IL-4, and IL-10 showed the same trends as for mRNA expression. To sum up, MenSC transplantation showed significant suppression in inflammation in IUA uterus; PRP amplified this effect. However, single PRP injection presented inflammation suppression trend but without significance.

Human gene expression was also analyzed between the two groups. Transcription products of secretory protein IGF-1 (insulin-like growth factor-1), SDF-1 (stromal cell-derived factor-1), and TSP-1 (thrombospondin-1) were found in M group and significantly augmented in MP group (Fig. 3g). To sum up, these results implied that the observed restoration of endometrium might be due to soluble paracrine factors released by MenSCs.

### Differentially expressed mRNA screening

**Fig. 4:**
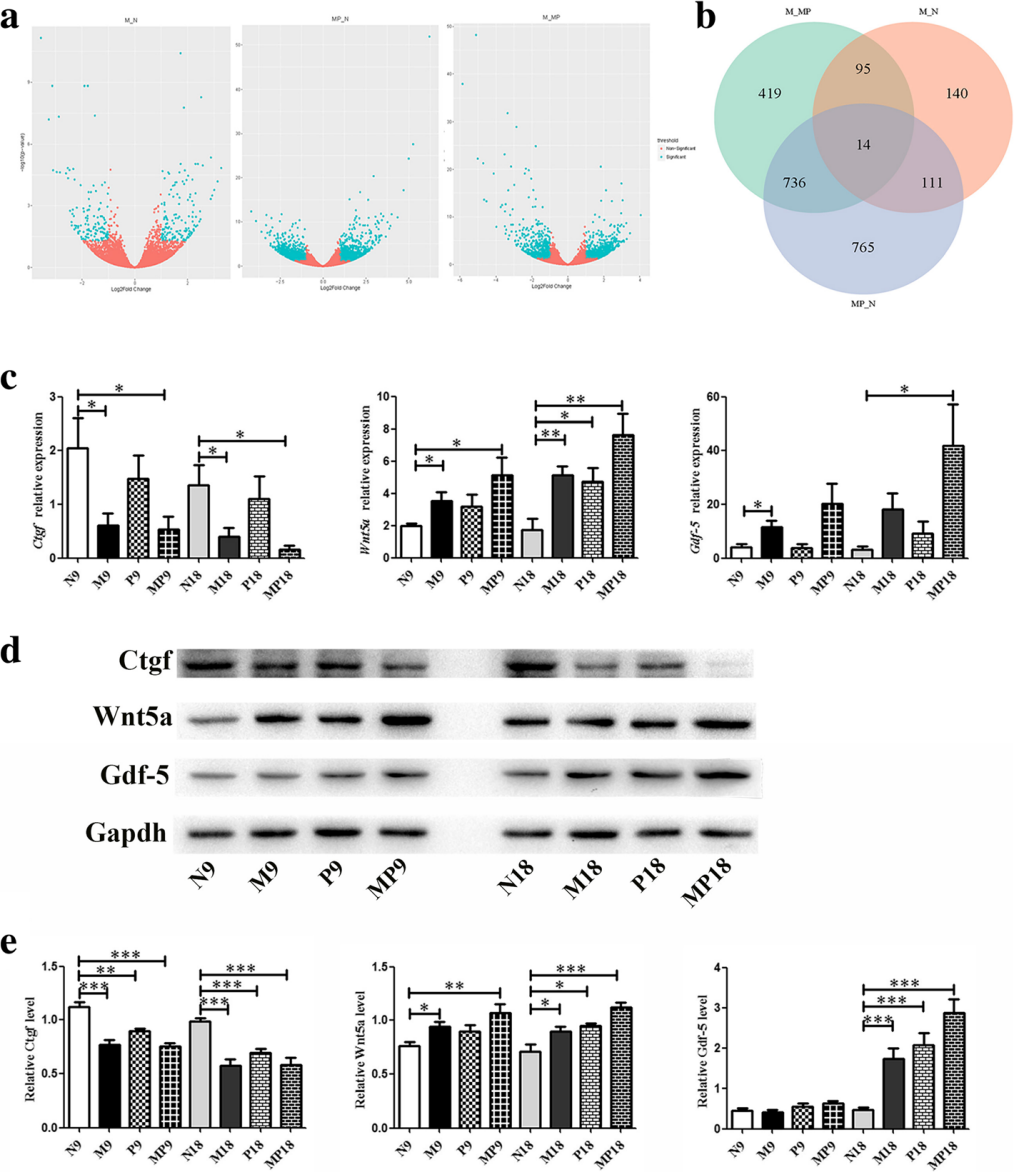
RNA sequencing analysis of deferentially expressed genes, verification of deferentially expressed proteins. **a** DESeq2 results in Volcano plot between N, M and MP groups (blue: differentially expressed genes, orange: non-differentially expressed genes). **b** Differentially expressed genes between comparison groups in Venn diagram. **c** Real-time PCR analysis of mRNA expression of Ctgf, Wnt5aand Gdf-5 in four treatment groupsat day 9 and day 18 (N≥6). **d** Western blot analysis of protein expression of Ctgf, Wnt5a and Gdf-5 in four treatment groupsat day 9 and day 18. **e** Statistical analysis of Western blot (N≥6)

To identify genes involved in MenSCs or MenSCs + PRP transplantation, three samples of each N group, M group, and MP group were analyzed using RNA sequencing. The samples were clustered according to the gene expression level. Differential gene expression analysis demonstrated that the transcriptome was distinguished between groups (Fig. 4a). Totally, 360 mRNAs were found to be differentially expressed between N and M group, including 186 genes that were significantly upregulated and 174 genes that were significantly downregulated. In addition, up to 1626 mRNAs were found to be differentially expressed between N and MP group, including 798 that were significantly upregulated and 828 that were significantly downregulated. Moreover, 125 mRNAs simultaneously changed both in M and MP groups (Fig. 4b). GO analysis indicated the simultaneously differentially expressed genes of N vs M group and N vs MP group were mainly enriched for regulation of cell proliferation, developmental processes (tissue development and system development), response to stimulus and other biological processes. The tissue development was mainly concentrated in muscle tissue development, connective tissue development, and epithelium development. The system development mainly concentrated on the vasculature, circulatory system, cardiovascular system, and skeletal system development. Pathway analysis indicated that the most significant changed pathway consisted of the Hippo signaling pathway, which is reported to be involved in regulating endometrial physiology and tissue fibrosis [20].

### Change of molecules of the Hippo signaling pathway

Three differentially expressed genes were selected to be analyzed in MenSCs and PRP-regulated restoration of intrauterine adhesions. As verified by real-time PCR (Fig. 4c), *Ctgf* was significantly downregulated by MenSCs and combined MenSCs + PRP at day 9 and day 18 post-transplantation. In addition, *Wnt5a* was significantly upregulated by MenSCs and PRP transplantation, while *Gdf-5* was remarkably increased at day 18 by MenSCs + PRP transplantation.

Ctgf is induced by TGF-β and is considered a downstream mediator of the effects of TGF-β on fibroblasts. TGF-β and Ctgf showed strong expression in the endometrium of the IUA patients [21]. In this study, protein expression analysis confirmed that MenSCs and MenSCs + PRP transplantation significantly inhibited Ctgf level, while Wnt5a and Gdf-5 were significantly increased in the uterus of M, P, and MP group (Fig. 4d, e).

## Discussion

This study demonstrated that the combination of MenSC and PRP transplantation was more effective in enhancing endometrial regeneration, compared to MenSCs or PRP alone in rat models with IUA. The combination therapy had a stronger effect on endometrial growth. Also, the pregnancy rate was much higher with the combined application of MenSCs and PRP. We also illustrated the mechanism of MenSCs in regulating uterine damage restoration. Increased proliferation of stromal cells, progenitor cells, and vessel density of rat endometrium implied that MenSCs affected rat stem cell differentiation and angiogenesis through paracrine effects. These results indicated that the MenSCs in combination with PRP transplantation was effective in treating refractory endometrium damage.

MenSCs has its distinguished advantages in regenerative medicine. Compared with mesenchymal stem cells (MSCs) isolated from other origins such as the umbilical cord, adipose tissue, and decidual tissue, MenSCs resemble most closely to BMSCs and have the highest clonogenic efficacy, the shortest population doubling time, and the largest number of in vitro passages before becoming senescent [22]. In recent years, the application of MenSCs in different kinds of disease has been thoroughly described [10, 23, 24].^.^Altogether, these findings pointed out that menstrual blood represents an efficient and ethically accepted source of MSCs for therapy. In a preliminary clinical trial, MenSCs have been used allogeneically to treat four patients with multiple sclerosis intravenously and intrathecally. The investigators reported that there was no evidence of immunological reactions or treatment-associated adverse effects after more than 1 year [25].

The abundance of therapeutic molecules rich in PRP are beneficial for cell proliferation and injury restoration [26]. In addition, the establishment of a PRP-containing scaffold for stem cell transplantation can significantly improve the therapeutic efficiency of MSCs [27]. In vitro, 5% PRP significantly promoted proliferation rate and increased expression of studied genes in bovine endometrial cells. According to MG’s results, in the conjunction of PRP and MSCs, it is inclined toward promoting the cellular proliferation of MSCs in vitro [28]. Recently, PRP application was proposed to improve expression of adhesion molecules, which was beneficial for endometrial receptivity [29, 30].

Our rodent results demonstrated that MenSCs had multiple effects on tissue recovery in the treatment of IUA. The locally injected MenSCs was mainly implanted in the reproductive tract, due to the strong homing ability to injured tissues. MenSCs have also been found to aggressively engraft into the portal areas of host liver following transplantation through tail vein in mice with CCl_4_-induced acute hepatic failure [9]. MenSCs are chemoattracted toward SDF-1, which binds to the specific receptor CXCR4 and has an important role in controlling stem cell recruitment [31]. Detection of human SDF-1 in MenSC transferred rats implied that an autocrine signal mediated the retention of stem cells in the uteri. Also, the augmentation of SDF-1 expression in MP group further enhanced MenSC homing to damaged endometrium, which elucidated the amplified function of transplantation combined with PRP. Recent studies have shown that MenSC-secreted growth factors are involved in ovarian function recovery in mice [32]. We speculated that growth factors transmitted paracrine signals were important in MenSC-treated endometrium. IGF-1 has been reported to be crucial for endometrial growth and receptive functions [33], while PRP treatment could enhance IGF-1 production [34]. Thus, the increased IGF-1 expression in MP group uteri might partly explain the significantly increased cellular proliferation in rats.

Inflammation has been suggested to hinder the recovery of injured endometrium and proven to be an important procedure involved in the deposition of fibrotic tissue in intrauterine adhesions [35]. Simultaneously, transcription factor nuclear factor-kappaB (NF-κB), which promotes the expression of inflammatory cytokines and plays an essential role in inflammatory diseases, was confirmed to be significantly elevated in endometrial samples from IUA patients and animal model [36]. On the other hand, MenSC transplantation has been investigated in the treatment of animals and clinical diseases associated with aberrant immune response [31, 37]. In the host, the MenSCs could attenuate the inflammation induction by reducing myeloperoxidase activity and could modulate the inflammation by downregulating IL-1β and upregulating IL-10 [37]. In this study, following the MenSC transplantation, TSP-1 expression was detected, and it increased with PRP addition. TSP-1 promoted the resolution of the inflammatory process and facilitated phagocytosis of damaged cells. Enhanced expression of TSP-1 could be a compensatory mechanism for controlling the immune response and protecting the tissues from excessive damage [38]. Accordingly, we suggest that MenSC transplantation contributed to endometrial regeneration partly through prevention of excessive inflammatory response.

Several studies demonstrated that PRP provides the effect of inflammation inhibition in mating-induced endometritis [39, 40]. In our previous study, the supplement of PRP in short-term culture reduced the expression of IL-6 in MenSCs in vitro [17]. However, in this study, our results showed that the PRP group provided a trend of inflammation inhibition, but without significance. According to our findings, the use of PRP alone may be not sufficient to treat severe intrauterine adhesion though it can significantly promote endometrial regeneration. Our results suggested that PRP has an excellent stem cell-assisted effect. Therefore, for clinical IUA patients, the combination with MenSCs was considered for patients with IUA in clinical.

Connective tissue growth factor (CTGF) is a secreted matricellular protein that regulates several aspects of cellular functions including proliferation, differentiation, survival, migration, adhesion, and stimulation of extracellular matrix production [41]. It has shown to be expressed in response to tissue injury and is considered as a prognostic marker in fibrotic diseases. CTGF is also a potential candidate in anti-fibrotic therapy approaches. Fibrotic signaling mediated by CTGF has been reported to be modulated by EGFR, TGF-β, or NF-κB signaling pathway [42–44]. In patients with AS, CTGF has been reported to be overexpressed in endometrial tissue [21, 45] and has been identified as therapeutic as well as prognosis indicator of treatment [19]. We have validated the increase of fibrosis in the endometrium by Masson and collagen I staining of rats in untreated N group. MenSC transplantation significantly reduced collagen deposition. Consistent with these results, mRNA and protein expressions of CTGF were downregulated. PRP application alone also suppressed fibrosis and decreased CTGF protein levels. A prospective synergy effect was found in MP group. However, the signaling pathway involved in MenSC-regulated CTGF expression still required further exploration. Wnt5a expression has been associated with endometrial gland development [46]. In vitro, Wnt5a promoted human embryonic stem cell differentiation into endometrium-like cells [47]. Our results showed that MenSCs and PRP significantly improved Wnt5a expression in the uterus, which could be the initiating factor of endometrial regeneration from stem cell differentiation. Increased Gdf-5 expression was involved in the decidualization of the endometrium, which is critical for implantation and the establishment of pregnancy [48]. Thus, MenSCs in combination with PRP transplantation might enhance the implantation rate in rats through upregulation of Gdf-5.

There are some limitations in the present study that need to be pointed out: (1) The leading function of MenSCs in the regeneration of endometrium should be identified to ensure the safety and efficacy of the therapy. As exosomes have been suggested to mediate the paracrine effects of MSCs [49], we plan to clarify the function of MenSC exosomes in IUA treatment by replacing the cell therapy with exosomes to avoid any risks of immune rejection and tumorigenesis. Moreover, as for the clinical application, (1) in a few patients from whom menstrual blood is difficult to acquire, MenSC exosomes from healthy donors could be an alternative choice; (2) signaling pathway involved in MenSCs that interacted with endometrium needs to be further explored in order to generalize the application of cell transplantation; gene editing of the differently expressed molecules including CTGF-, Wnt5a-, and Gdf-5-associated regulators in MenSCs needs to be conducted to identify the key factors in the recovery of endometrium; (3) furthermore, randomized controlled trial should be conducted to confirm the therapeutic effect of MenSCs and PRP in patients with IUA.

## Conclusions

In conclusion, we showed the effectiveness of combination therapy with MenSCs and PRP in the treatment of rat IUA. The underlying mechanism of endometrial restoration appears to be multifaceted. In addition to cellular proliferation, migration, and differentiation, inflammation was also regulated by transplanted MenSCs. Overall, MenSCs is a valuable source of cells for transplantation in the treatment intrauterine adhesion. Combined with PRP, this cell therapy was more effective.

## Additional files

Additional file 1: **Table S1.** Quantitative polymerase chain reaction primer sequences. (DOCX 17 kb)

Additional file 2: **Figure S1.** GFP labeling of MenSCs. (JPG 155 kb)

Additional file 3: **Figure S2.** (A) DiI labeling of MenSCs. (B) The location of DiI-MenSCs in uteri after injected into the uterus at day 9. (JPG 213 kb)

## Abbreviations

b-FGF: Basic fibroblast growth factor
BMSCs: Bone marrow-derived stem cells
CK-18: Cytokeratin-18
CTGF: Connective tissue growth factor
IGF: Insulin-like growth factor
IUA: Intrauterine adhesion
IUD: Intrauterine devices
MenSCs: Menstrual blood-derived stromal cells
MSCs: Mesenchymal stem cells
PDGF: Platelet-derived growth factor
PRP: Platelet-rich plasma
SDF-1: Stromal cell-derived factor-1
TGF-β1: Transforming growth factor-β1
TSP-1: Thrombospondin-1
VEGF: Vascular endothelial growth factor
