# Supplementary material for: Platelet-rich plasma improves therapeutic effects of menstrual blood-derived stromal cells in rat model of intrauterine adhesion
Source: Stem Cell Res Ther. 2019 Feb 15;10:61. doi: 10.1186/s13287-019-1155-7 (PMC6377773; doi:10.1186/s13287-019-1155-7)
Supplement: Supplementary file 1 — Table S1. Quantitative polymerase chain reaction primer sequences. (DOCX 17 kb) [file 13287_2019_1155_MOESM1_ESM.docx]

Additional file 1: **Table S1** Quantitative polymerase chain reaction primer sequences

| Gene Name Sequence (5′-3′) |
| --- |
| **IL1-β F: CAGCAGCATCTCGACAAGAG**  **R: AAAGAAGGTGCTTGGGTCCT**  **IL4 F: TGTACCTCCGTGCTTGAAGA**  **R: TTGTGAGCGTGGACTCATTC**  **IL6 F: AGTTGCCTTCTTGGGACTGA**  **R: CCTCCGACTTGTGAAGTGGT**  **IL10 F: GAAGCTGAAGACCCTCTGGA**  **R: TGGCCTTGTAGACACCTTTG**  **IGF-1 F: CAGCAGTCTTCCAACCCAAT**  **R: ACAGCGCCAGGTAGAAGAGA**  **SDF-1 F: AGAGCCAACGTCAAGCATCT**  **R: CCACTTTAGCTTCGGGTCAA**  **TSP-1 F: TGTGACATGTGGTGATGGTG**  **R: GTCTTTCTTGCAGGCTTTGG**  **Collagen1 F: ATCTCCTGGTGCTGATGGAC**  **R: AGACCAGGGAAGCCTCTTTC**  **Ctgf F: GCTGCACCAGTGTGAAGACC**  **R: ACTTGAACTCCACCGGCAGT**  **Wnt 5a F: CATGGAGTGTCTGGCTCCTG**  **R: CGCTGCGCTGTCATACTT**  **GDF-5 F: TCCACTGCGAAGGACTGTGT**  **R: TCCACTGCGAAGGACTGTGT**  **GAPDH(rat) F: TGGTGAAGGTCGGTGTGAAC**  **R: GACTGTGCCGTTGAACTTGC**  **GAPDH(human) F: CAGGAGGCATTGCTGATGAT**  **R: GAAGGCTGGGGCTCATTT** |
