# Supplementary figures and images for: Platelet-rich plasma improves therapeutic effects of menstrual blood-derived stromal cells in rat model of intrauterine adhesion
Source: Stem Cell Res Ther. 2019 Feb 15;10:61. doi: 10.1186/s13287-019-1155-7 (PMC6377773; doi:10.1186/s13287-019-1155-7)

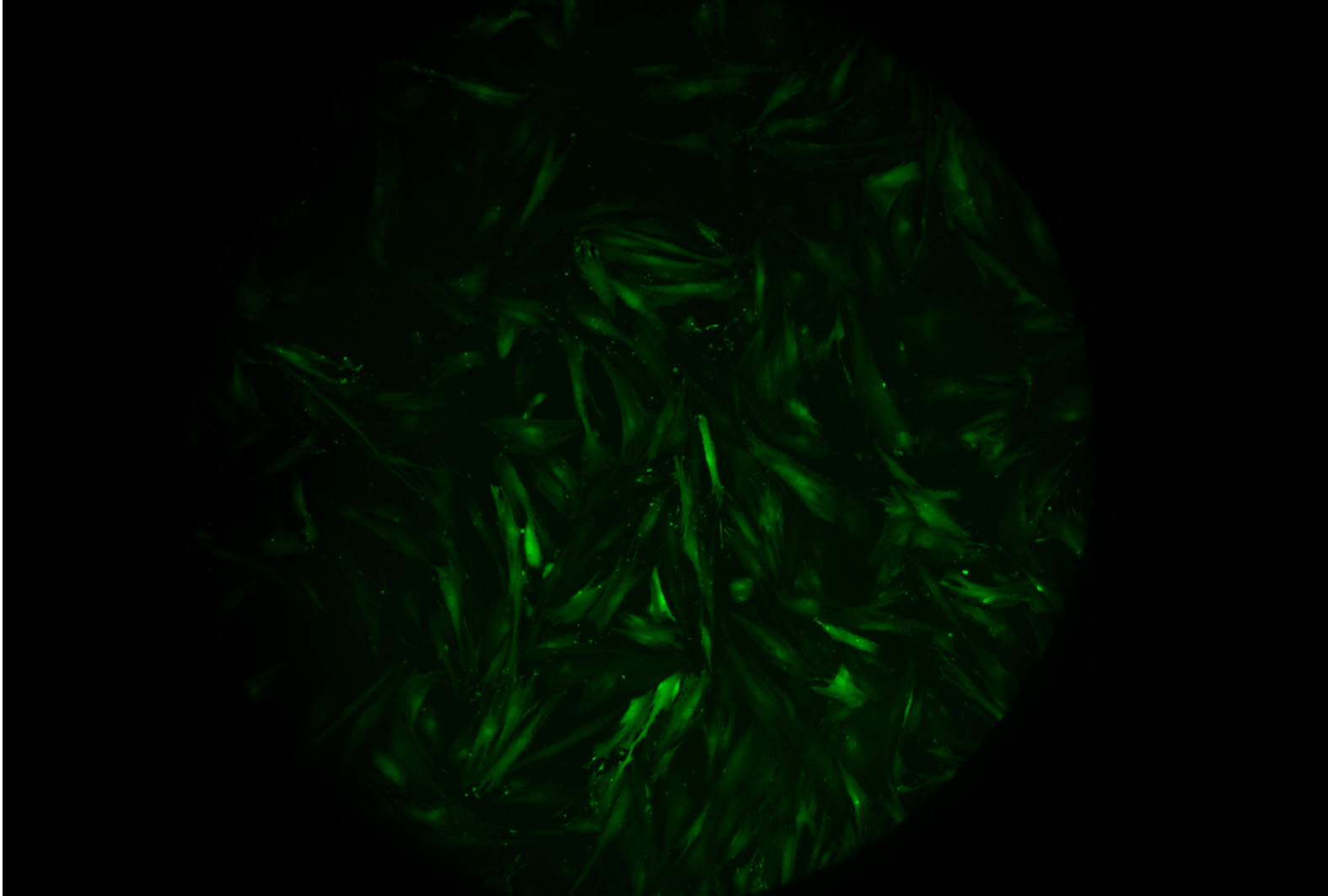

Supplement: Supplementary file 2 — Figure S1. GFP labeling of MenSCs. (JPG 155 kb) [file 13287_2019_1155_MOESM2_ESM.jpg]

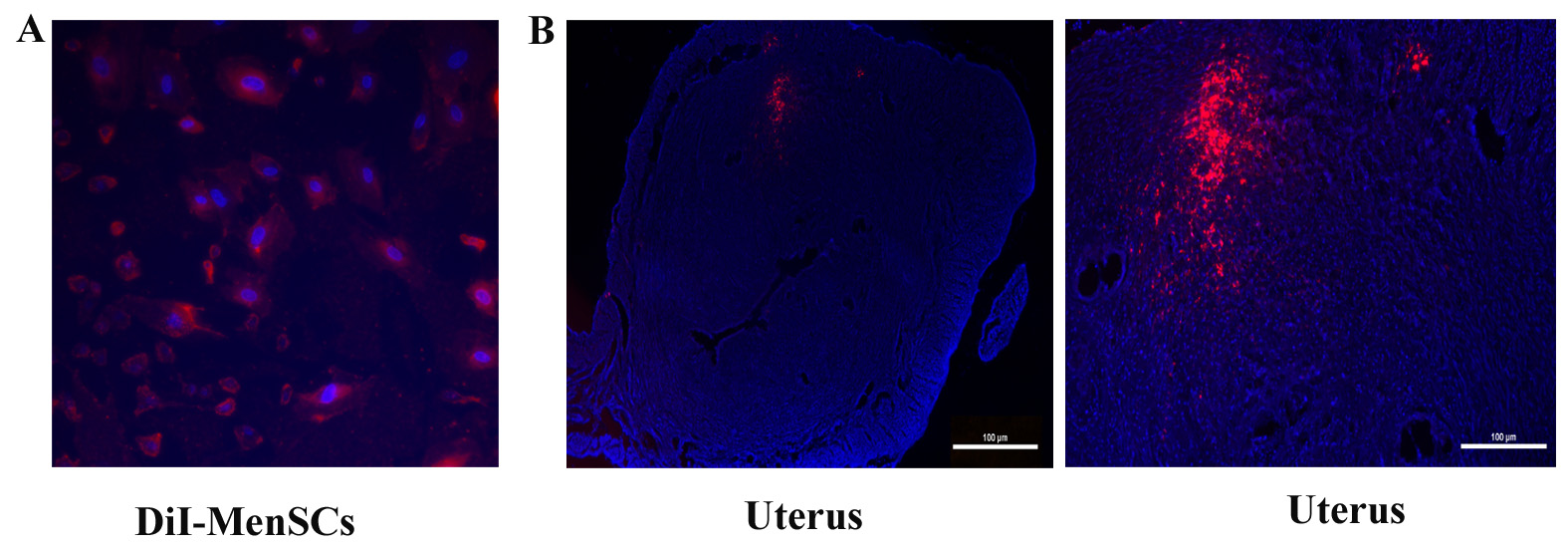

Supplement: Supplementary file 3 — Figure S2. (A) DiI labeling of MenSCs. (B) The location of DiI-MenSCs in uteri after injected into the uterus at day 9. (JPG 213 kb) [file 13287_2019_1155_MOESM3_ESM.jpg]
